# *In Vitro* and *In Vivo* Efficacy of Monepantel (AAD 1566) against Laboratory Models of Human Intestinal Nematode Infections

**DOI:** 10.1371/journal.pntd.0001457

**Published:** 2011-12-27

**Authors:** Lucienne Tritten, Angelika Silbereisen, Jennifer Keiser

**Affiliations:** 1 Department of Medical Parasitology and Infection Biology, Swiss Tropical and Public Health Institute, Basel, Switzerland; 2 University of Basel, Basel, Switzerland; Queensland Institute for Medical Research, Australia

## Abstract

**Background:**

Few effective drugs are available for soil-transmitted helminthiases and drug resistance is of concern. In the present work, we tested the efficacy of the veterinary drug monepantel, a potential drug development candidate compared to standard drugs *in vitro* and in parasite-rodent models of relevance to human soil-transmitted helminthiases.

**Methodology:**

A motility assay was used to assess the efficacy of monepantel, albendazole, levamisole, and pyrantel pamoate *in vitro* on third-stage larvae (L3) and adult worms of *Ancylostoma ceylanicum*, *Necator americanus* and *Trichuris muris*. *Ancylostoma ceylanicum*- or *N. americanus-*infected hamsters, *T. muris*- or *Ascaris suum*-infected mice, and *Strongyloides ratti*-infected rats were treated with single oral doses of monepantel or with one of the reference drugs.

**Principal Findings:**

Monepantel showed excellent activity on *A. ceylanicum* adults (IC_50_ = 1.7 µg/ml), a moderate effect on *T. muris* L3 (IC_50_ = 78.7 µg/ml), whereas no effect was observed on *A. ceylanicum* L3, *T. muris* adults, and both stages of *N. americanus*. Of the standard drugs, levamisole showed the highest potency *in vitro* (IC_50_ = 1.6 and 33.1 µg/ml on *A. ceylanicum* and *T. muris* L3, respectively). Complete elimination of worms was observed with monepantel (10 mg/kg) and albendazole (2.5 mg/kg) in *A. ceylanicum*-infected hamsters. In the *N. americanus* hamster model single 10 mg/kg oral doses of monepantel and albendazole resulted in worm burden reductions of 58.3% and 100%, respectively. *Trichuris muris*, *S. ratti and A. suum* were not affected by treatment with monepantel *in vivo* (following doses of 600 mg/kg, 32 mg/kg and 600 mg/kg, respectively). In contrast, worm burden reductions of 95.9% and 76.6% were observed following treatment of *T. muris*- and *A. suum* infected mice with levamisole (200 mg/kg) and albendazole (600 mg/kg), respectively.

**Conclusions/Significance:**

Monepantel reveals low or no activities against *N. americanus*, *T. muris*, *S. ratti and A. suum in vivo*, hence does not qualify as drug development candidate for human soil-transmitted helminthiases.

## Author Summary

Soil-transmitted helminthiases affect more than one billion people among the most vulnerable populations in developing countries. Currently, control of these infections primarily relies on chemotherapy. Only five drugs are available, all of which have been in use for decades. None of the drugs are efficacious using single doses against all soil-transmitted helminths (STH) species and show low efficacy observed against *Trichuris trichiura*. In addition, the limited availability of current drug treatments poses a precarious situation should drug resistance occur. Therefore, there is great interest to develop novel drugs against infections with STH. Monepantel, which belongs to a new class of veterinary anthelmintics, the amino-acetonitrile derivatives, might be a potential drug candidate in humans. It has been extensively tested against livestock nematodes, and was found highly efficacious and safe for animals. Here we describe the *in vitro* and *in vivo* effect of monepantel, on *Ancylostoma ceylanicum*, *Necator americanus*, *Trichuris muris*, *Strongyloides ratti*, and *Ascaris suum*, five parasite-rodent models of relevance to human STH. Since we observed that monepantel showed only high activity on one of the hookworm species and lacked activity on the other parasites tested we cannot recommend the drug as a development candidate for human soil-transmitted helminthiases.

## Introduction

The hookworm species *Ancylostoma duodenale* and *Necator americanus*, the whipworm *Trichuris trichiura*, the threadworm *Strongyloides stercoralis*, and the roundworm *Ascaris lumbricoides* are soil-transmitted helminths (STH) of great public health importance. Cumulatively, these parasites affect more than one billion people globally, particularly in developing regions of Asia, Africa, and Latin America [1], [2]. If untreated, infections with STH are present for years and patients suffer from moderate to severe intestinal disturbances, anemia, nutrient loss and profound physical and mental deficiencies [3], [4].

Helminth control relies primarily on the regular administration of anthelmintics, typically carried out within the framework of school-based deworming programs, once or twice a year [5]–[7]. Five drugs are currently available for the treatment of infections with STH (albendazole, mebendazole, pyrantel pamoate, levamisole, and ivermectin), all of which have been registered for human use before or during the 1980's [8], [9]. No new anthelmintic drug for human use has reached the market since then. Moreover, none of these drugs are efficacious using single doses on all STH species, with particularly low efficacy observed on *T. trichiura* [10]. Relying on only a handful of drugs is a precarious situation, in the light of a possible emergence of drug resistance [10].

Since drug resistance to nematodes of veterinary importance is widely spread and increasing in frequency, most of the anthelmintic drug research and development efforts are motivated by veterinary needs [11]. For example, albendazole, mebendazole, and pyrantel pamoate were originally developed for livestock and pets [12].

Monepantel (AAD1566) belongs to a new class of veterinary anthelmintics, the amino-acetonitrile derivatives. It has been proposed that monepantel interferes with nematode-specific acetylcholine receptor subunits, leading to body wall muscle paralysis and subsequent death of worms. Due to its unique mode of action, the drug has proven efficacy against nematodes infecting livestock which are resistant to current anthelmintic drugs [13]. Monepantel has been extensively tested on different nematode isolates. Administered at a single oral dose of 2.5 mg/kg, it was found to be safe, well-tolerated by ruminant hosts, and showed high cure rates on fourth stage larvae and adult worms of 15 nematode species [13]–[16]. Due to its high and broad nematocidal activity, monepantel was considered to be a candidate for a human health directed program.

The aim of the present investigation was to study the activity of monepantel, compared with the reference drugs, albendazole, levamisole, and pyrantel pamoate, in five parasite-rodent models, that correspond to important human STH and *in vitro*. *Ancylostoma ceylanicum* and *Necator americanus* were both adapted using eggs from infected dogs or humans to an unnatural host, the golden hamster, and represent robust rodent models for hookworm infections [17], [18]. The *Trichuris muris* mouse model is an excellent model for trichuriasis [19], [20]. *Strongyloides ratti* in rats is a commonly used murine model for strongyloidiasis [21]. Finally, murine infection with *Ascaris suum* is a model which mimics the early infection of *A. lumbricoides*. The survival of different larval stages and adult worms of *A. ceylanicum*, *N. americanus*, and *T. muris* was evaluated *in vitro*, following monepantel incubation using a motility assay. The *in vitro* activity of monepantel on *S. ratti* has been described recently [22]. *In vivo*, we studied worm burden reductions and for *A. ceylanicum*, *N. americanus*, and *T. muris*, worm expulsion rates were also measured.

## Methods

### Drugs

Monepantel was kindly provided by Novartis Animal Health, St-Aubin, Switzerland. Albendazole and pyrantel pamoate were purchased from Sigma-Aldrich (Buchs, Switzerland), and levamisole-hydrochloride from Fluka (Buchs, Switzerland).

For the *in vitro* studies, stock solutions of the drugs were prepared in 100% DMSO (Fluka, Buchs, Switzerland) and stored at 4°C. For the *in vivo* studies, drugs were suspended in 7% (v/v) Tween 80% and 3% (v/v) ethanol or DMSO/PEG shortly before treatment.

### Animals and Parasites

Three-week-old male Syrian Golden hamsters were purchased from Charles River (Sulzfeld, Germany). Four-week-old female NMRI mice and 3-week-old female C57Bl/6J mice were purchased from Harlan (Horst, The Netherlands). Three-week-old female Wistar rats were purchased from Harlan (Horst, The Netherlands).

All animals were kept in macrolon cages under environmentally-controlled conditions (temperature: 25°C, humidity: 70%, light/dark cycle 12 h/12 h) and had free access to water and rodent food (Rodent Blox from Eberle NAFAG, Gossau, Switzerland). They were allowed to acclimatize in the animal facility of the Swiss Tropical and Public Health Institute (Swiss TPH) for 1 week before infection. The current study was approved by the local veterinary agency based on Swiss cantonal and national regulations (permission no. 2070).

### Parasites and Infections

*Ancylostoma ceylanicum* third-stage larvae (L3) were kindly provided by Prof. J. M. Behnke (University of Nottingham). The *A. ceylanicum* life cycle [23] had been maintained at the Swiss TPH since June 2009 [17]. To maintain the life cycle, hamsters were treated orally 1 day before infection and then twice weekly with 3 mg/kg hydrocortisone (Hydrocortone®, MSD) or with 1 mg/l dexamethasone (dexamethasone water-soluble, Sigma-Aldrich) in the drinking water. They were orally infected with 150 *A. ceylanicum* L3, which had been harvested less than 1 month before infection and had been assessed microscopically for viability. Animals assigned to *in vivo* studies were not treated with hydrocortisone and were infected with 300 L3.

Infective *N. americanus* L3 were the gift of Prof. S. H. Xiao (National Institute for Parasitic Diseases, Shanghai). Hamsters were immunosuppressed with dexamethasone as described above and were infected subcutaneously with 250 viable *N. americanus* L3.

Embryonated *T. muris* eggs were kindly obtained from Prof. J. M. Behnke and Prof. H. Mehlhorn. The life cycle had been maintained at the Swiss TPH since January 2010 as described elsewhere [19]. Briefly, *T. muris* eggs were evaluated for embryonation under the microscope (magnification 80–160×, Carl Zeiss, Germany). NMRI mice were orally infected with 400 embryonated eggs. Mice were treated either subcutaneously (s.c.) 1 day before infection and then every second day between days 5 and 15 with 15 mg hydrocortisone (Hydrocortisone 21-hemisuccinate sodium salt, Sigma-Aldrich) in 0.9% NaCl solution, or with 8 mg/l dexamethasone in the drinking water until the end of the experiment.

The *S. ratti* life cycle had been maintained over decades at the Swiss TPH, by serial passage through rats. Rats were infected subcutaneously with 735 freshly harvested *S. ratti* L3.

Infective *A. suum* eggs were obtained from Prof. S. M. Thamsborg, University of Copenhagen and Prof. G. Cringoli, University of Naples. Briefly, C57Bl/6J mice were orally infected with 500 embryonated eggs, according to a procedure described elsewhere [24].

### *In vitro* Studies

The larval or adult motility assay is currently the method of choice to evaluate drug sensitivity of different nematode species [25]–[27]. Non-motile worms were considered as dead and the percent viability or survival in each well was calculated.

### *A. ceylanicum* and *N. americanus*

#### Activity against L3

Thirty L3 in 100 µl deionized water, supplemented with antibiotics, were placed in each well of a 96-well plate (Costar). The L3 had been harvested less than 1 month before the studies and were stored in deionized water containing 25 µg/ml amphotericin B (Sigma-Aldrich) and 1% (v/v) penicillin-streptomycin solution (10,000 U/ml penicillin and 10 mg/ml streptomycin, Sigma-Aldrich). Drugs were serially-diluted in HBSS (Gibco) supplemented with 25 µg/ml amphotericin B (Sigma Aldrich) and 1% (v/v) penicillin-streptomycin solution (10,000 U/ml penicillin and 10 mg/ml streptomycin, Sigma-Aldrich). Diluted drug (100 µl; 100–0.01 µg/ml, final concentration) were added to the wells and the plate was incubated for 72 h at room-temperature in a dark and humid box. Larval motility was evaluated under the microscope (magnification 20×) following addition of 100 µl hot water (∼90°C) and exposure to microscope light. A minimum of 2 wells served as controls, which were L3 incubated with the highest concentration of DMSO used in the test (2% v/v).

#### Activity against Adults

Drug susceptibility on adults was tested in 48-well plates (Costar). Three to 4 worms were added to wells containing 500 µl supplemented HBSS medium, containing 10% v/v fetal calf serum (Gibco). Drug dilutions (500 µl) were added and the plate was incubated for 72 h at 37°C and 5% CO_2_. The motility of adult worms was evaluated under the microscope (magnification 20×), after adding 500 µl hot water (∼90°C), using a viability scale (2: good motility, 1: lowered motility, and 0: no motility, death). A minimum of 3 worms served as controls which were incubated in the presence of the highest concentration of DMSO used in the test (2% v/v).

#### Ovicidal Activity

The drug effects on eggs were observed using a modified protocol of the egg hatch test [28]. Stools of infected hamsters were collected overnight, filtered and subjected to flotation by mixing the stool with a 2 M NaNO_3_ solution and centrifuged at 2000 rpm. The upper quarter of the solution (containing the eggs) was kept and washed twice in deionized water. The test was carried out in quadruplicate for each drug concentration. Fifty eggs in 500 µl deionized water were distributed to each well and 500 µl drug diluted in deionized water were added (10 µg/ml, final concentration). After 24 and 48 h, 20 eggs per well were examined under the microscope (magnification 80–160×) for embryonation and hatching, respectively.

### *T. muris*

#### Activity against L3 and Adults

L3 or adults were collected from the intestines of infected mice and 3–4 worms were distributed to each well of 48 or 96-well plates (Costar), containing 100 µl (L3) or 500 µl (adults) RPMI medium supplemented with 5% (v/v) amphotericin B (Sigma Aldrich) and 1% (v/v) penicillin-streptomycin solution (10,000 U/ml penicillin and 10 mg/ml streptomycin, Sigma-Aldrich). Drug dilutions (100 µl for testing on L3 or 500 µl for adults) ranging from 200–50 µg/ml, final concentration, were added and the plate was incubated for 72 h at 37°C and 5% CO_2_. The larval and adult motilities were evaluated under the microscope (magnification 20–80×) using a viability scale (3: good motility, 2: low motility, 1: very low motility, and 0: death), as described by Stepek and colleagues [27]. A minimum of 3 worms served as controls, which were incubated in the highest concentration of DMSO used in the test (2% v/v).

### *In vivo* Studies

#### *A. ceylanicum* and *N. americanus*

The immunosuppressive treatment of hamsters was stopped at least 2 days before treatment. Hamsters were housed individually from day 20 post-infection (p.i.) onwards. On days 21 and 22 (*A. ceylanicum*) or 46 and 47 (*N. americanus*), a fecal sample was collected from each hamster and processed using an in-house sedimentation method. Briefly, stools were collected overnight, soaked in 0.9% NaCl solution, filtered and washed with 150 ml saline through 8 gauze layers. The filtrate was allowed to sediment for 1–2 h and the solution adjusted to 0.1 g filtrate per ml. To calculate the number of eggs per gram (epg), the average of 4 egg counts of 20 µl of fecal solution was determined microscopically. On the basis of the fecal egg burden, *A. ceylanicum*-infected hamsters were assigned to the following treatment groups - monepantel 10 mg/kg or 5 mg/kg, albendazole 5 mg/kg, 2.5 mg/kg or 1.25 mg/kg, levamisole 10 mg/kg or pyrantel pamoate 10 mg/kg- or control groups, with 4 animals per group. Similarly, groups of 3–4 *N. americanus*-infected animals received 20 or 10 mg/kg monepantel, 10 or 5 mg/kg albendazole, or served as controls.

Hamsters were treated on day 23 p.i. (*A. ceylanicum*) or 48 p.i. (*N. americanus*) with single oral doses of the test drugs. Expelled worms were counted from the collected stools 24 h and 48 h after treatment. On day 7 post-treatment, the hamsters were killed by the CO_2_ method and the remaining worms in the gut counted [29]–[31].

#### *T. muris*

Treatment with dexamethasone was stopped at least 2 days before treatment. Mice were housed individually from day 40 onwards. A fecal sample was examined from each mouse and egg negative animals were excluded from the study. Groups of 4 infected animals were assigned to treatment (monepantel 600 mg/kg, albendazole 600 mg/kg, levamisole 200 mg/kg, or pyrantel pamoate 300 mg/kg) or control groups. Expelled worms were counted from the collected stools 24 h and 48 h after treatment. On day 7 post-treatment, mice were killed by the CO_2_ method and the remaining worms in the gut counted [30].

#### *S. ratti*

Five days p.i, 1 group of 4 rats was treated orally with 32 mg/kg monepantel. Four rats were left untreated and served as controls. Seven days post-treatment, the rats were dissected. The intestine was removed, opened and incubated for 3 h at 37°C in PBS, as described before [32]. The liquid and the intestines were screened under a binocular, and the larvae counted (magnification 10–40×).

#### *A. suum*

One hour p.i, 2 groups of 4 mice were treated with single oral doses of 600 mg/kg monepantel or 600 mg/kg albendazole. A third group of 4 mice was left untreated and served as control. On day 7 post-treatment, the mice were killed by the CO_2_ method. The lungs and livers were removed, cut with fine scissors, and incubated in 0.9% NaCl for 24 h at 37°C to allow larvae to migrate out of the organs into the saline [24]. Larvae in solution were counted using a microscope (magnification 20×).

### Statistical Analyses

The average of motility scores for one drug was calculated for each concentration and normalized into percentage, relative to control. IC_50_ values were expressed based on the median effect principle using CompuSyn (version 1.0). The r value represents the linear correlation coefficient of the median-effect plot, indicating the goodness of fit, hence the accuracy of the IC_50_ [33]. Variance analysis in the ovicidal activity studies was performed with the Fisher's exact test, using StatsDirect (version 2.4.5; StatsDirect Ltd; Cheshire, UK). The worm burden reductions were determined by comparing the mean number of adult worms in the intestine of a treated group with the mean numbers of worms in the control group. Means and standard deviations were calculated using Microsoft® Excel 2003. The worm expulsion rates were calculated by dividing the number of expelled worms of a treatment group by the group's total worm burden. The Kruskal-Wallis test and the Mann-Whitney U test were used to assess the statistical significance of the worm burden reduction, using StatsDirect.

## Results

### *In vitro* Findings

The effects of monepantel and reference drugs on L3 and adult worms of *A. ceylanicum* and *T. muris* after 72 h of exposure *in vitro* are presented in Table 1.

**Table 1:** 50% inhibitory concentrations of monepantel, albendazole, levamisole, and pyrantel pamoate on *A. ceylanicum*, *N. americanus*, and *T. muris*.

Drugs	*A. ceylanicum*		*N. americanus*		*T. muris*	
	IC_50_ in µg/ml (r)		IC_50_ in µg/ml (r)		IC_50_ in µg/ml (r)	
	L3	Adults	L3	Adults	L3	Adults
Monepantel	>100 (0.70)	1.7 (0.93)	>100 (n.d.)	>100 (0.32)	78.7 (1.0)	>200 (n.d.)
Albendazole	32.4 (0.87)	>100 (0.77)	>100 (n.d.)	>100 (0.57)	>200 (0.39)	>200 (0.40)
Levamisole-HCl	1.6 (0.95)	>100 (0.79)	0.5 (0.97)	13.4 (0.99)	33.1 (1.0)	16.5 (1.0)
Pyrantel pamoate	90.9 (0.84)	>100 (0.79)	2.0 (0.94)	7.6 (0.98)	95.5 (0.99)	34.1 (0.99)

IC_50_s (µg/ml) were calculated for monepantel, albendazole, levamisole, and pyrantel pamoate after 72 h on L3 and adult stages of *A. ceylanicum*, *N. americanus*, and *T. muris*. r = linear correlation coefficient of the median-effect plot, indicating the goodness of fit. r≥0.85 indicates a satisfactory fit. n.d.: not determined, fitting not possible.

#### *A. ceylanicum*

The sensitivity of *A. ceylanicum* L3 to monepantel was low at the highest concentration tested (100 µg/ml), with over 70% of the larvae still showing activity after 72 h following stimulation with hot water and exposure to light (IC_50_>100 µg/ml). Pyrantel pamoate affected the larvae only moderately (IC_50_ = 90.9 µg/ml, r = 0.84), whereas an IC_50_ of 32.4 µg/ml was calculated for albendazole (r = 0.87). Levamisole showed superior efficacy: at a concentration of 10 µg/ml a survival rate of 26.7% was determined. An IC_50_ value of 1.6 µg/ml (r = 0.95) was calculated for levamisole (Table 1).

*Ancylostoma ceylanicum* adult worms were highly sensitive to monepantel at concentrations of 1 µg/ml and above (IC_50_ = 1.7 µg/ml, r = 0.93). Lower activities against adult *A. ceylanicum* were observed for albendazole, levamisole, and pyrantel pamoate, which showed survival rates of 60–95% at 100 µg/ml (all IC_50_s>100 µg/ml) (Table 1).

As shown in Table 2, albendazole was the only compound that inhibited both egg embryonation and hatching *in vitro* (90.5% and 98.5% inhibition of embryonation and hatching, respectively). Levamisole, pyrantel pamoate, and monepantel showed no effect on embryonation and only a moderate inhibition on hatching (reduction of 4.7–41.5%).

**Table 2:** Ovicidal activity of monepantel, albendazole, levamisole, and pyrantel pamoate on *A. ceylanicum* eggs.

Group	% Embryonation at 24 h (SD)	% Hatching at 48 h (SD)
Control	100 (3.5)	100 (9.8)
Monepantel	99.5 (9.3)	68.6 (9.2)*
Albendazole	9.5 (5.2)*	1.5 (3.0)*
Levamisole-HCl	88.6 (8.2)*	58.5 (3.5)*
Pyrantel pamoate	95.4 (7.0)	95.3 (12.5)

SD = standard deviation.

*P-value <0.001 (Fisher's exact test).

#### *N. americanus*

Neither monepantel nor albendazole had an effect on the viability of larvae or adult *N. americanus* (Table 1). Levamisole was found the most efficacious drug at both stages, with calculated IC_50_s of 0.5 µg/ml (r = 0.97) against larvae and 13.4 µg/ml (r = 0.99) against adults. Both stages were found to be sensitive to pyrantel pamoate (IC_50_s = 2.0 µg/ml, r = 0.94 and 7.6 µg/ml, r = 0.98, respectively).

#### *T. muris*

A reduction in viability of *T. muris* L3 was observed following exposure to monepantel (IC_50_ = 78.7 µg/ml, r = 1.0), whereas adult worms were not affected (IC_50_>200 µg/ml). Albendazole showed no activity against *T. muris* L3 or adults *in vitro* (IC_50_>200 µg/ml). The highest activity on *T. muris* L3 and adults *in vitro* was observed with levamisole (IC_50_s = 33.1 µg/ml, r = 1.0, and 16.5 µg/ml, r = 1.0, respectively) followed by pyrantel pamoate (IC_50_s = 95.5 µg/ml, r = 0.99 and 34.1 µg/ml, r = 0.99, respectively) (Table 1).

### *In vivo* Findings

#### *A. ceylanicum*

The worm expulsion rates and worm burden reductions determined for monepantel, albendazole, levamisole, and pyrantel pamoate administered to *A. ceylanicum*-infected hamsters at single oral doses are shown in Table 3. Treatment with monepantel at 10 mg/kg resulted in complete elimination of the worms. At a dose of 5 mg/kg a worm expulsion rate of 42.7% and a worm burden reduction of 56.8% were observed. The worm burden reductions in monepantel-treated hamsters were statistically significant (*P* = 0.046). Treatment with albendazole at 5 and 2.5 mg/kg cured all *A. ceylanicum*-infected hamsters. At a dose of 1.25 mg/kg the worm expulsion rate and worm burden reduction were 70.5% and 87.8%, respectively. There was a highly significant difference between the worm burden of albendazole-treated hamsters (1.25–5 mg/kg) and control hamsters (*P*<0.001). Moderate activities were observed for levamisole (worm burden reduction of 60.2%, *P* = 0.057) and pyrantel pamoate (worm burden reduction of 87.2%, *P* = 0.057 and worm expulsion rate of 63.4%) administered at 10 mg/kg.

**Table 3:** Dose response relationships of monepantel, albendazole, levamisole, and pyrantel pamoate on *A. ceylanicum in vivo*.

Group	Dose(mg/kg)	Mean number of worms (SD)	Mean number of expelled worms (SD)	Worm expulsion rate (%)	Worm burden reduction (%)	*P*-value
Control 1	–	29.5 (21.2)	0	0	–	–
Control 2	–	24.5 (8.8)	0	0	–	–
Control 3	–	29.7 (4.2)	0.3 (0.6)	1.1	–	–
Control 4	–	27.0 (4.0)	0.3 (0.6)	1.2	–	–
Monepantel	5^1^	22.3 (10.1)	9.5 (4.4)	42.7	56.8	0.046*
	10^1^	16.0 (9.7)	16.0 (9.7)	100	100	
Albendazole	1.25^4^	11.0 (6.9)	7.8 (3.8)	70.5	87.8	<0.001*
	2.5^3^	6.3 (7.1)	6.3 (7.1)	100	100	
	5^2^	6.3 (7.1)	6.3 (7.1)	100	100	
Levamisole-HCl	10^2^	17.5 (7.2)	7.8 (2.5)	44.3	60.2	0.057§
Pyrantel pamoate	10^3^	10.3 (9.3)	6.5 (6.2)	63.4	87.2	0.057§

SD = standard deviation. The numbers in superscript refer to the corresponding control group.

*Kruskal Wallis test comparing the median of the worm burdens of control and treated hamsters (all doses versus control),

**§:** Mann-Whitney U-test comparing the median of the worm burdens of control and treated hamsters (one dose versus control).

#### *N. americanus*

As presented in Table 4, no dose-response relationship could be observed following administration of monepantel to *N. americanus*-infected hamsters. Both worm expulsion rate and worm burden reduction were 58.3% after treatment with a single dose of 10 mg/kg monepantel, whereas a worm expulsion rate and worm burden reduction of 38.6% and 0%, respectively were obtained following treatment with 20 mg/kg. The worm burden reductions in monepantel-treated hamsters were not statistically significant (*P* = 0.830). In comparison, albendazole administered at 5 mg/kg achieved a worm expulsion rate of 69.6% and a worm burden reduction of 70.8%. A complete elimination of worms was observed at 10 mg/kg albendazole (*P* = 0.028).

**Table 4:** Effects of monepantel and albendazole on *N. americanus in vivo*.

Group	Dose(mg/kg)	Mean number of worms (SD)	Mean number of expelled worms (SD)	Worm expulsion rate (%)	Worm burden reduction (%)	*P*-value*
Control	–	8.0 (7.5)	0 (0)	0	–	–
Monepantel	20	19.0 (12.0)	7.3 (4.5)	38.6	0	0.830
	10	8.0 (4.4)	4.7 (3.1)	58.3	58.3	
Albendazole	10	6.7 (2.5)	6.7 (2.5)	100	100	0.028
	5	7.7 (3.1)	5.3 (3.8)	69.6	70.8	

SD = standard deviation.

*Kruskal Wallis test comparing the median of the worm burdens of control and treated hamsters (all doses versus control).

#### *T. muris*

The worm expulsion rates and worm burden reductions determined for monepantel, albendazole, levamisole, and pyrantel pamoate administered to *T. muris*-infected mice at single oral doses are shown in Table 5. A single dose of 200 mg/kg levamisole resulted in a worm expulsion rate of 90.5% and a worm burden reduction of 95.9% (*P* = 0.036). Albendazole (600 mg/kg) achieved a worm expulsion rate of 49.4% and a worm burden reduction of 20.2%. No effect was observed with pyrantel pamoate (300 mg/kg) and monepantel (600 mg/kg) (worm expulsion rate and worm burden reduction <10%).

**Table 5:** Effects of monepantel, albendazole, levamisole, and pyrantel pamoate on *T. muris in vivo*.

Group	Dose(mg/kg)	Mean number of worms (SD)	Mean number of expelled worms (SD)	Worm expulsion rate (%)	Worm burden reduction (%)	*P*-value§
Control	–	90.0 (41.5)	1.0 (1.2)	0	–	–
Monepantel	600	106.6 (159.1)	1.7 (0.6)	1.6	0	0.536
Albendazole	600	140.3 (116.6)	69.3 (48.1)	49.4	20.2	0.536
Levamisole-HCl	200	29.0 (60.9)	26.3 (54.6)	90.5	95.9	0.036
Pyrantel pamoate	300	282.3 (246.6)	26.7 (20.6)	9.4	0	0.095

SD = standard deviation.

**§:** Mann-Whitney U test comparing the median of the worm burdens of control and treated mice.

#### *S. ratti*

*S. ratti*-infected rats treated with 32 mg/kg monepantel showed a worm burden reduction of 0% compared to the untreated control group (*P* = 0.77).

#### *A. suum*

Monepantel displayed negligible effect on the worm burden in the mice's lungs and liver, 7 days after treatment with 600 mg/kg (worm burden reduction = 3.3%, *P* = 0.886, Table 6), compared to the control animals. Albendazole administered at the same dose achieved a worm burden reduction of 76.6% (*P* = 0.171).

**Table 6:** Effects of monepantel and albendazole on *A. suum in vivo*.

Group	Dose(mg/kg)	Mean number of worms following treatment (SD)	Worm burden reduction (%)	*P*-value§
Control	–	48.4 (37.33)	–	–
Monepantel	600	46.8 (21.33)	3.3	0.886
Albendazole	600	11.35 (13.26)	76.6	0.171

SD = standard deviation.

**§:** Mann-Whitney U test comparing the median of the worm burdens of control and treated mice.

## Discussion

To date, only five drugs are included in the WHO model list of essential medicines to treat infections with human STH. Most of these anthelmintics were discovered before the 1980s. Though there is no evidence yet for emerging resistance to any of these drugs in human helminth populations, there are worrying signs that anthelminthic efficacy may be declining [34], [35]. In addition, the increased frequency of reported low cure rates, in particular against *T. trichiura* and hookworm infections, highlight the need to find alternative drugs [10].

*A. ceylanicum*, *N. americanus*, *T. muris*, *S. ratti*, and *A. suum* are five well-established laboratory parasite-rodent models of relevance to human STH. The aim of the present study was to determine their sensitivities to monepantel, a broad spectrum and safe drug used for livestock which recently entered the market for veterinary use. It is one of the few available drug candidates eligible for rapid transitioning into development for human STH infections [36].

Monepantel activates signaling *via* nematode-specific DEG-3 subtype nicotinic acetylcholine receptors (nAChRs), causing a hypercontraction of the body wall muscles leading to paralysis and hence, death of the worm [13]. ACR-23 protein, a member of the DEG-3 group in *Caernohabtitis elegans*, and its homolog MPTL-1 in *Haemonchus contortus*, another model for gastrointestinal nematodes, are major targets of monepantel. The absence of MPTL-1, observed in some nematode species resulted in reduced drug sensitivity [22].

*Ancylostoma ceylanicum* adult worms were found to be highly sensitive to monepantel *in vitro*, in contrast to the third-stage larvae, but the drug lacked ovicidal activity. Hamsters harboring adult *A. ceylanicum* were cleared from the worms following a 10 mg/kg single oral dose of monepantel. *N. americanus* was not affected by the drug *in vitro* and only moderately susceptible *in vivo* to 10 mg/kg or higher doses. These findings suggest a relative stage and species specificity, which might be explained by the absence of a functional MPTL-1 homolog in *A. ceylanicum* L3 and possibly in L3 and adult *N. americanus*.

*Trichuris muris* third-stage larvae were only moderately sensitive to monepantel after incubation for 72 h *in vitro*, whereas adult stages were not affected, neither *in vitro* nor *in vivo*. Monepantel had already been reported to lack activity against *T. ovis*, *a* minor parasite of sheep [15], [16], a finding that is in accordance with our data.

In addition, monepantel lacked activity in *S. ratti*-infected rats, a result in line with a recent investigation, which revealed that *S. ratti* third-stage larvae were not affected by monepantel after 72 h of incubation [22]. For comparison, a complete elimination of adult worms was achieved with ivermectin (0.5 mg/kg) in *S. ratti*-infected rats [32]. *Strongyloides ratti* has a remote homolog of DES-2 and ACR-23/MPTL-1 only, which is not targeted by monepantel [22].

Finally, although only one high dosage was tested, our data indicate that *A. suum* is not affected by treatment with monepantel *in vivo*, whereas albendazole reduced the worm burden of *A. suum* in mice at the same dose.

Like *H. contortus*, *A. ceylanicum* and *N. americanus* are members of the nematode clade V, whereas *S. ratti* belongs to clade IV, *A. suum*, to clade III, and *T. muris* to clade I [37]. One could hypothesize that only clade V species exhibit sensitivity to monepantel, whereas those that diverged from this lineage of the evolutionary tree earlier (clades I to IV) might not have evolved homologous receptors. As available for *A. suum* [38], further genome sequencing of *A. ceylanicum*, *N. americanus* and *Trichuris* spp. remains to be performed in order to extend current knowledge about evolutionary and functional relationships of receptors involved in sensitivity to monepantel.

In the present investigation, albendazole, levamisole, and pyrantel pamoate have been extensively studied *in vitro* and *in vivo*. The results obtained are in agreement with earlier *in vivo* [29], [39]–[41] and *in vitro* [41] work using *A. ceylanicum* and *N. americanus*. In addition, to our knowledge, the *in vitro* and *in vivo* sensitivities of these three drugs against *T. muris* are presented for the first time. In line with human efficacy data [10], albendazole showed highly potent activity against *A. ceylanicum*, *N. americanus* and *A. suum*, yet much less pronounced activity against *T. muris in vivo*. A similar trend was observed for pyrantel pamoate, which achieved a moderate effect against *A. ceylanicum* but lacked activity in *T. muris*-infected mice. These results on pyrantel pamoate are compatible with cure rates reported in clinical trials [10]. On the other hand, levamisole was highly efficacious in our ancylostomiasis and trichuriasis rodent models, while low to moderate cure rates have been recently reported in humans [10], [42].

Interestingly, contradictory results were obtained with albendazole, levamisole, and pyrantel pamoate against adult *A. ceylanicum in vitro* and *in vivo*. In addition, albendazole showed excellent activity in the *N. americanus* hamster model but lacked activity *in vitro*. This finding might be partially explained by the presence of active metabolites, since for example albendazole and levamisole are rapidly metabolized *in vivo* [43]–[45]. In addition, large differences in sensitivity between larval and adult hookworm stages were observed with levamisole (and albendazole for *A. ceylanicum*). It is commonly accepted that the benzimidazoles tend to be lethal to developing stages but not always to adult worms. Developing cells are obviously more harmed by the benzimidazoles, as the utilization of tubulin in the mitotic cycles is affected [46].

In conclusion, to our knowledge, we have for the first time analyzed the efficacy of monepantel in animal models corresponding to human intestinal helminthiases. A recently developed target product profile suggested that a drug development candidate for the treatment of infections with STH should ideally target all stages (at least adult and ova) and species of the major geohelminths such as *Ascaris*, *Trichuris*, both hookworm species and *Enterobius* [36]. Hence, based on our results, established in nematode-rodent models, monepantel does not fulfill the required minimal product characteristics for a new intestinal anthelmintic.
